# HLA Loci and Recurrence of Focal Segmental Glomerulosclerosis in Pediatric Kidney Transplantation

**DOI:** 10.1097/TXD.0000000000001201

**Published:** 2021-08-26

**Authors:** Brian I. Shaw, Alejandro Ochoa, Cliburn Chan, Chloe Nobuhara, Rasheed Gbadegesin, Annette M. Jackson, Eileen T. Chambers

**Affiliations:** 1 Department of Surgery, Duke University, Durham, NC.; 2 Department of Biostatistics and Bioinformatics, Duke University, Durham, NC.; 3 School of Medicine, Duke University, Durham, NC.; 4 Department of Pediatrics, Duke University, Durham, NC.; 5 Department of Immunology, Duke University, Durham, NC.

## Abstract

Recurrent focal segmental glomerulosclerosis (FSGS) after kidney transplantation accounts for the majority of allograft failures in children with primary FSGS. Although current research focuses on FSGS pathophysiology, a common etiology and mechanisms of disease recurrence remain elusive.

**Methods.:**

We performed a retrospective review of the Scientific Registry of Transplant Recipients to determine the association of specific HLA recurrence of FSGS. Kidney transplants recipients under the age of 19 who were diagnosed with FSGS, who were transplanted after January 1, 2000, and who had complete HLA data were included in the study. We performed simple logistic regression on all HLA A, B, C, DR, and DQ represented in the dataset and FSGS recurrence and then determined those associated with recurrence using the Benjamini–Hochberg method for multiple comparisons. For those HLAs that were associated with recurrence, we further determined the effect of matching recipient and donor HLA with recurrence.

**Results.:**

HLA DR7, DR53, DQ2, DR52, and DQ7 were associated with increased or decreased risk of recurrent disease after transplantation. We identified a risk haplotype consisting of HLA-DR7, DR53, and DQ2 that was consistently associated with an increased risk of recurrence (odds ratio 1.91; 95% confidence interval, 1.44-2.54, *P* < 0.001). We also found that donor/recipient concordance for HLA-DQ7 was associated with a decreased risk of recurrence (odds ratio 0.42; 95% confidence interval, 0.37-0.53, *P* = 0.009).

**Conclusions.:**

HLA profiles may be used for risk stratification of recurrence of FSGS in pediatric kidney transplant recipients and deserves further study.

Posttransplant recurrence of focal segmental glomerulosclerosis (FSGS) occurs in up to 50% of first-time kidney transplant recipients and can be challenging to treat.^1,2^ Early posttransplant FSGS recurrence is characterized by nephrotic syndrome (NS) with massive proteinuria within hours to days and progression to acute tubular necrosis, delayed graft function,^3^ and early allograft loss.^4^ Posttransplant FSGS recurrence accounts for the majority of allograft failures in children transplanted for primary FSGS.^5^ The return to dialysis, sensitization for future transplants, and increased risk of recurrence for subsequent allografts become particularly problematic for pediatric recipients.^6^ Additionally, posttransplant FSGS results in significant recipient morbidity and mortality, places a substantial financial burden on the healthcare system, and contributes to the ongoing organ shortage.

Currently, adequate pretransplant risk assessment for developing posttransplant FSGS recurrence is lacking and patients have variable responses to therapy following recurrence.^7-9^ Risk factors for FSGS recurrence have included living donation,^10-12^ younger age,^11,13^ initial steroid sensitivity,^11^ White race,^12^ and histologic characteristics (mesangial proliferation and minimal change disease) on initial native kidney biopsy and late steroid resistant nephrotic syndrome.^14^ These risk factors, however, have been inconsistent across studies. As we and others and have found associations between HLA class II alleles and recurrence of primary NS, it is plausible that specific HLA may also confer risk of disease recurrence following kidney transplantation.^15-24^ and represent an opportunity to predict posttransplant FSGS risk.

In this analysis, we sought to determine if specific HLAs were associated with posttransplant FSGS recurrence in pediatric recipients. We first identified recipient HLA associated with primary FSGS and then explored the relationship of recipient and donor HLA with FSGS recurrence posttransplant.

## MATERIALS AND METHODS

### Data Source and Study Population

We used data from the Scientific Registry of Transplant Recipients (SRTR). This study was IRB approved and determined exempt (Pro00106450). The SRTR data system includes data on all donors, waitlisted candidates, and transplant recipients in the United States. These data are submitted by the members of the Organ Procurement and Transplantation Network. The Health Resources and Services Administration, US Department of Health and Human Services provides oversight to the activities of the Organ Procurement and Transplantation Network and SRTR contractors. To enrich our cohort for patients with primary NS, we examined the recurrence rate of patients whose primary indication for transplantation was focal glomerulosclerosis or FSGS and included only pediatric patients (age <18 y). We excluded those with potential secondary causes of FSGS, such as diabetes, congenital anomalies of kidney and urinary tract, and other glomulerulonephritides.

**FIGURE 1.**
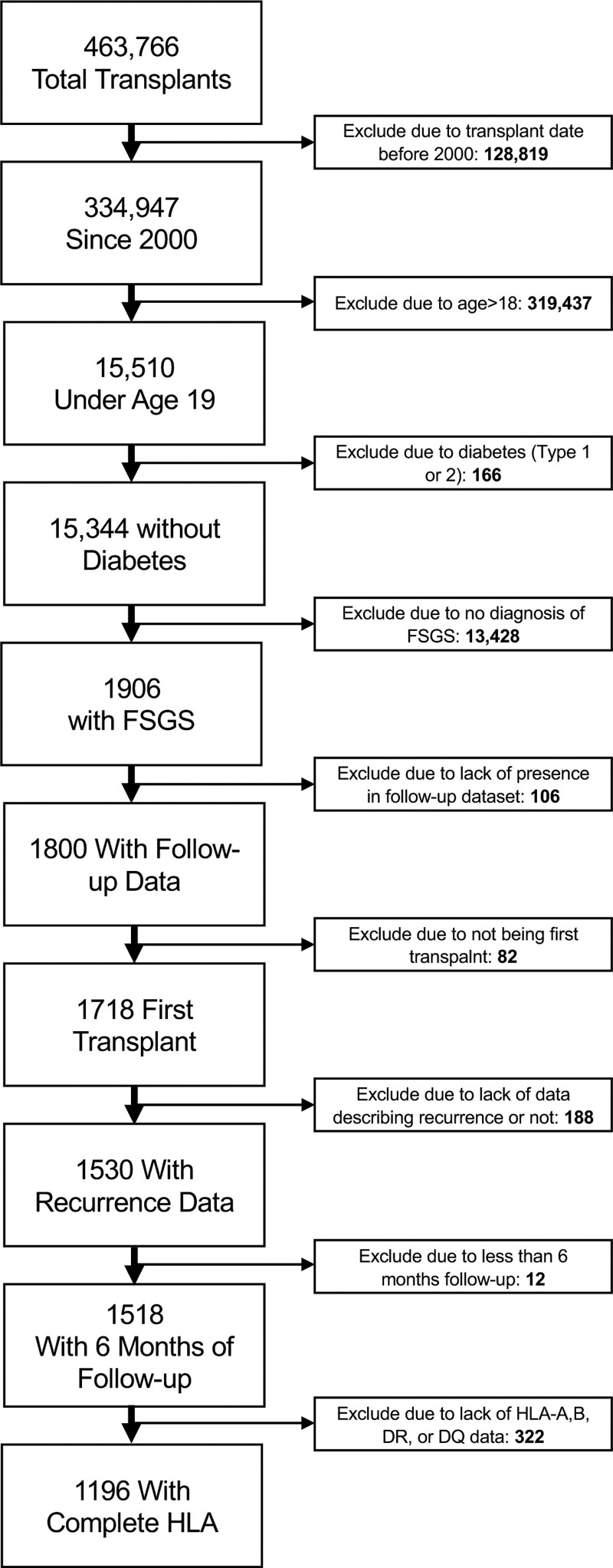
Cohort definition flow diagram. FSGS, focal segmental glomerulosclerosis.

Therefore, all patients who underwent kidney transplant after 1999 (334 947), who were listed by age 18 (15 510), who did not have diabetes (15 344), who were diagnosed with focal glomerulosclerosis or FSGS (1906), who underwent kidney alone transplant (1901), who were in the follow-up dataset (txf_ki, 1800), who were undergoing their first transplant (1718), who had data on recurrence (1530) with at least 6 mo of follow-up (1512), and who had complete recipient HLA-DR and HLA-DQ information (final N = 1196) were included in this analysis (Figure 1).

### Primary and Secondary Outcomes

Our primary outcome was recurrence of their primary disease (FSGS) as reported by the individual transplant centers. Secondary outcomes examined graft and patient survival time and time to disease recurrence.

### Statistical Analyses

Patient and donor characteristics were summarized and compared using chi-squared test for categorical variables and Wilcoxon Rank-Sum tests for continuous variables as appropriate using 2-tailed *P* values. Of note, a single donor could contribute 2 kidneys, and therefore, the number of donors and recipients are not exactly equal. Univariable logistic regression was performed to determine the association of an HLA antigen (A, B, C, DR51, DR52, DR53, DR, and DQ) with disease recurrence using likelihood ratio tests and 2-tailed *P* values. Of note, generally only serological typing is reported to UNOS. When molecular data were available, they were converted to serologic types for consistency.^25^ For all recipient HLA loci except HLA-C, complete serologic data were available for all HLA types. As the association of class I HLA was an exploratory analysis, we performed a complete case analysis for HLA-C associations.

After serial univariable logistic regression across all HLA antigens in the dataset, we tested for significance using the Benajamini–Hochberg method with a false discovery rate of 20%. For all HLA that were significantly correlated at this point, we performed a second set of logistic regression analyses in which we examined the dose effect (ie, being a heterozygote or homozygote for each HLA) of these HLA in the recipient and the risk of recurrence as well as the dose effect of the donor having a concordant HLA with the recipient. We performed a complete case analysis for both of these dose effect scenarios for each HLA (ie, we excluded recipients from the concordance analysis if their donor lacked HLA information).

We performed multiple regression to determine our ability to predict posttransplant recurrence. We performed model construction using factors previously postulated to increase the risk of NS recurrence after transplant (age of disease onset, living donor type, race, and ethnicity) as well as all HLA antigen level data (recipient, donor, and concordant) that were significantly correlated with recurrence. We assessed for collinearity by using correlation matrices and evaluation of the variance inflation factor. We chose our final model by examining the Akaike information coefficient, Bayesian information coefficient, and by examining likelihood ratio tests between models as less significant terms were removed. Receiver operator characteristics for the final models were calculated.

We also performed a time to event analysis for time to recurrence using the Kaplan–Meier method to plot curves and the Log-rank test to compare them.

A sensitivity analysis repeating the initial step of this analysis—performing simple logistic regression to determine the association between recurrence and HLA antigen—was repeated accounting for race (White versus non-White) and Latino ethnicity did not substantively change results. All analyses were performed using STATA v. 15 (Statacorp, College Station, TX).

## RESULTS

### Cohort Characteristics and Graft Failure Associated With FSGS Recurrence

**FIGURE 2.**
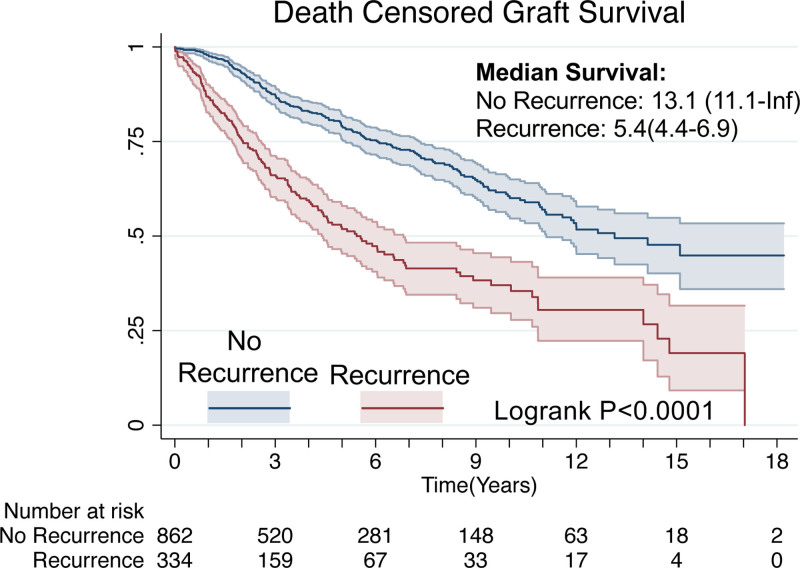
Kaplan–Meier curve of death censored graft survival. Comparison by Log-rank test.

Patient and donor characteristics are summarized in Tables 1 and 2, respectively. Our cohort consisted of 1196 patients. In the cohort as a whole, the median age at listing was 15 (interquartile range [IQR] 11–17), 43% were female, 58% were white, 36% were Black, 27% were Latino, and the median BMI was 20 (IQR 17–24). Overall, 28% of patients in our cohort experienced recurrence. Patients with posttransplant FSGS recurrence were younger (median [Med] age 14 [IQR] [10–17] versus 16 [12–18]), and had lower albumin (Med [IQR] 3.3 [2.4–3.8] versus 3.7 [3.2–4.2]) and body mass index (Med [IQR] 19.1 [17.0–23.0] versus 20.5 [17.5–24.8]). Additionally, recurrence was associated with earlier time to graft failure, with median death censored graft survival of 5.4 y in patients with recurrence compared to 13.1 y in patients without recurrence (Figure 2, Log-rank *P* < 0.001).

**TABLE 1.** Recipient characteristics

	No recurrence	Recurrence	*P*
	N = 862	N = 334	
Gender (female), n (%)	371 (43%)	146 (44%)	0.83
Age at transplant, Med (IQR)	16.0 (12.0–18.0)	14.0 (10.0–17.0)	**<0.001**
Age at listing, Med (IQR)	15 (12–17)	13 (9–16)	**<0.001**
Race, n (%)			0.20
Asian	27 (3%)	5 (1%)	
Black	323 (37%)	115 (34%)	
Multiracial	11 (1%)	6 (2%)	
Native American	5 (1%)	1 (0%)	
Pacific Islander	3 (0%)	4 (1%)	
White	493 (57%)	203 (61%)	
Ethnicity (Latino), n (%)	251 (29%)	79 (24%)	0.058
Time on dialysis (mo), Med (IQR)	15 (7–27)	16 (8–29)	0.42
Albumin (g/dL), Med (IQR)	3.7 (3.2–4.2)^*c*^	3.3 (2.4–3.8)^*c*^	**<0.001**
Body mass index, Med (IQR)	20.5 (17.5–24.8)^*b*^	19.3 (17.0–23.0)^*b*^	**0.002**

Bolded *P* are significant at *P* < 0.05 level.

^a^0%–5% Missing.

^*b*^5%–10% Missing.

^*c*^10%–20% Missing.

^d^>20% Missing.

IQR, interquartile range; Med, median.

**TABLE 2.** Donor characteristics

	No recurrence	Recurrence	*P*
	N = 856	N = 328	
Donor type (living), n (%)	217 (25%)	96 (29%)	0.17
Related donor, n (%)	170 (20%)	74 (23%)	0.30
Donor gender (female), n (%)	338 (39%)	136 (41%)	0.53
Donor age, MED (IQR)	25.0 (19.0–34.0)	26.5 (19.0–38.0)	0.12
Donor race, n (%)			0.18
Asian	13 (2%)	10 (3%)	
Black	135 (16%)	46 (14%)	
Multiracial	6 (1%)	0 (0%)	
Native American	9 (1%)	1 (0%)	
Pacific Islander	1 (0%)	0 (0%)	
White	692 (81%)	271 (83%)	
Body mass index, Med (IQR)	25.1 (22.2–28.8)	24.7 (21.5–28.5)	0.12
Inotrope support, n (%)	334 (54%)^*d*^	128 (56%)^*d*^	0.51
Cause of death, n (%)			0.044
Anoxia	135 (21%)^*d*^	55 (24%)^*d*^	
CVA	79 (12%)^*d*^	37 (16%)^*d*^	
Head trauma	416 (65%)^*d*^	133 (57%)^*d*^	
CNS tumor	3 (0%)^*d*^	0 (0%)^*d*^	
Other	6 (1%)^*d*^	7 (3%)^*d*^	
Donor diabetes, n (%)	5 (1%)	3 (1%)	0.48
Cigarette smoking, n (%)	64 (10%)^*d*^	23 (10%)^*d*^	0.97
Cocaine use, n (%)	73 (12%)^*d*^	27 (12%)^*d*^	0.92
Cold ischemia time (h), Med (IQR)	10 (5–16)^*b*^	9 (4–16)^*b*^	0.70

Bolded *P* are significant.

^*a*^0%–5% Missing.

^*b*^5%–10% Missing.

^*c*^10%–20% Missing.

^*d*^>20% Missing.

CNS, central nervous system; CVA, cerebrovascular accident; IQR, interquartile range; Med, median.

### Recurrence of FSGS After Transplant Is Predicted by Recipient HLA

**FIGURE 3.**
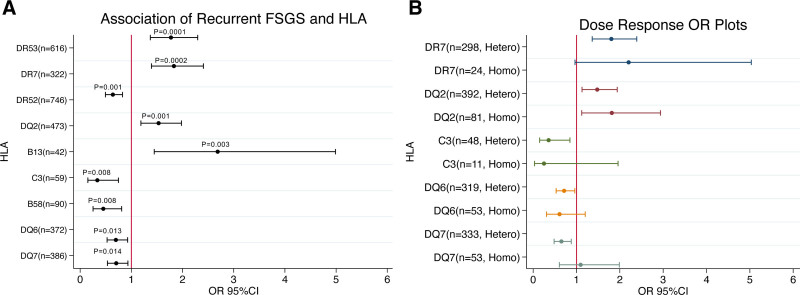
Association of HLA with FSGS recurrence. A, Alleles associated with risk of recurrence after utilizing the Benjamini–Hochberg correction with a false discovery rate of 20%. Odds ratios are for association with disease recurrence. Antigens are ordered from most to least significant in from top to bottom. Point estimates with 95% CI shown. B, Dose response of select Recipient HLA. Heterozygotes and homozygotes for each HLA shown. Odds ratios are for association with disease recurrence. Point estimates with 95% CI shown. HLA B13, B58, DR52, and DR53, not shown because of lack of information on heterozygosity or too few patients for modeling. CI, confidence interval; FSGS, focal segmental glomerulosclerosis; OR, odds ratio.

We examined the association of HLA antigens with recurrence at any time posttransplant. In Figure 3A, we see that HLA antigens HLA-B13, DR7, DQ2, and DR53 were most highly associated with recurrence risk, whereas HLA-C3, B58, DR52, DQ6, and DQ7 were associated with decreased rates of recurrence when controlling for a false discovery rate of 20%. Of note, we retained HLA-B13, DR7, DR52, and DR53 with a false discovery rate of 5%. We also investigated alleles associated with early recurrence (reported within the first y posttransplant) with a false discovery rate of 5% and obtained positive associations with B13, DR7, DR53, DQ2, and DQ8, and negative associations with DR52 and DQ6. All alleles except DQ8 were noted in our initial analysis. We next assessed a dose effect of certain antigens by determining the odds of recurrence given recipient heterozygosity or homozygosity (Figure 3B). In general, antigens showed a dose-response pattern, with homozygosity causing the point estimate to be more extreme in the same direction as the heterozygote, though this trend was not observed for DQ7. Note that B13, B58, DR52, and DR53 are excluded from this analysis either due to lack of data on heterozygosity or insufficient number of heterozygotes for analysis. Since homozygotes are less common than heterozygotes, there is less power to detect differences in association between those 2 dosages.

### Defining a Recipient HLA Risk Haplotype for FSGS Recurrence

**FIGURE 4.**
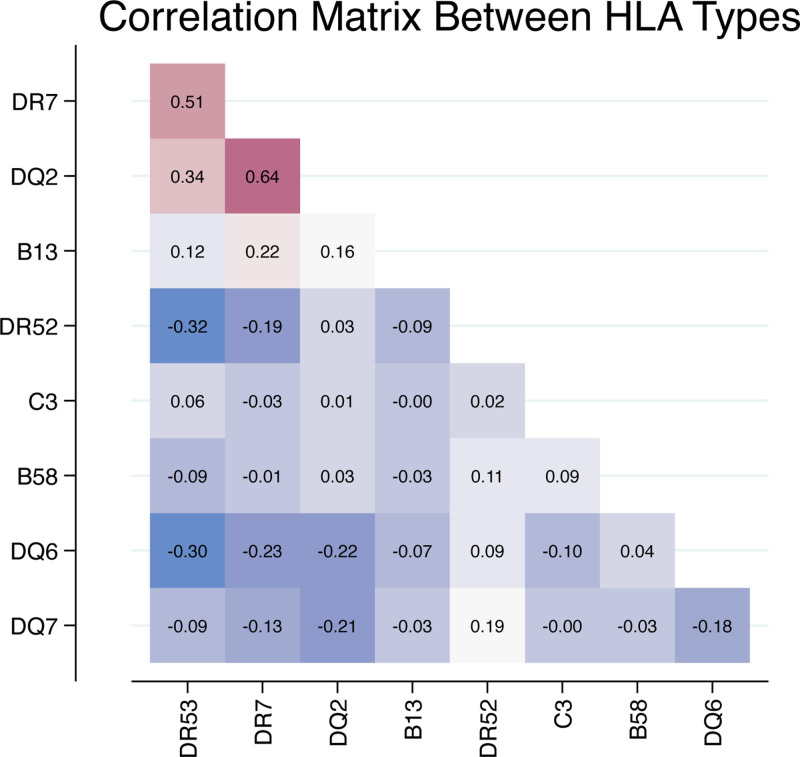
Association between all Recipient HLA antigens significantly associated with disease recurrence. HLA-DR7, DR53, and DQ2 are all positively correlated with one another. Pearson’s correlation coefficient shown.

Given that HLA-DR7, DR53, and DQ2 represent a common HLA haplotype^26^ and because we noted that these antigens were positively correlated with one another (Figure 4), we interrogated the association of this potential multi-HLA antigen recipient haplotype (now referred to as the risk haplotype) with recurrence. In a simple logistic regression analysis, the risk haplotype increased the risk of recurrence nearly 2-fold (odds ratio [OR] 1.91 95% confidence interval [CI] 1.44-2.54, *P* < 0.001). The risk haplotype also is associated with increased risk of early recurrence within the first year (OR 2.27; 95% CI 1.65-3.13). Expressed as raw percentages, 27% of those with the risk haplotype recurred within the first year, whereas among patients with the C3 allele, only 6% recurred in the same time period.

### Recipient HLA Antigens Are Associated With Time to FSGS Recurrence

**FIGURE 5.**
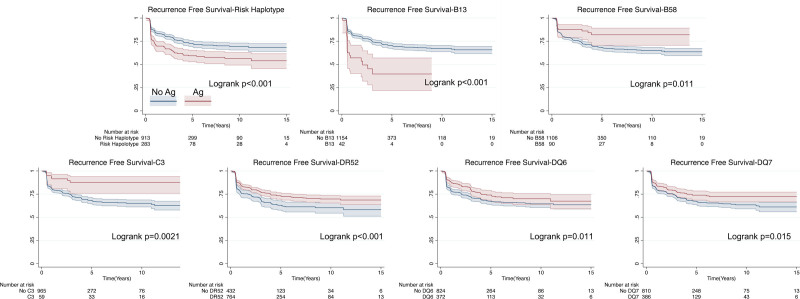
Kaplan–Meier curves for significantly correlated HLA. Blue curves are without the specified antigen and red curves are with the specified antigen. All comparisons by log-rank test.

We assessed whether our identified recipient HLA antigens were associated not only with a binary event of recurrence but also time to recurrence. Kaplan–Meier plots for each associated HLA antigen are shown in Figure 5. Overall, the results are similar to those shown in Figure 2, with a decrease in recurrence-free survival for the risk haplotype and B13 and an increase in recurrence-free survival for HLA-B58, C3, DR52, DQ6, and DQ7.

### Impact of Donor HLA on FSGS Recurrence

We also investigated a role for donor HLA in disease recurrence and potential dose effects when the donor shared a risk or protective HLA antigen with the recipient. The donor HLA alone did not appear to impact recurrence rate (data not shown); however, there was an apparent dose effect for recipient/donor pairs that shared DQ7. The proportion of patients that recurred was lower in recipient/donor pairs that shared the protective HLA-DQ7 antigen (13.3% for concordance versus 23.3% for the recipient allele alone). This effect was confirmed when performing multiple logistic regression using an interaction term between donor and recipient antigens (OR 0.42; 95% CI 0.37-0.53, *P* = 0.009). Under logistic regression, no other HLA antigens were observed to impact recurrence when shared between recipient and donor.

### Using HLA Allows for the Modest Overall Prediction of FSGS Recurrence

We next constructed a multivariable model for the prediction of disease recurrence (Table 3). Our initial model included all terms previously seen to be significant on univariable analysis including recipient HLA (the risk haplotype [HLA-DR7-DR53-DQ2], HLA-B13, DR52, C3, B58, DQ6, and DQ7), recipient/donor sharing of HLA-DQ7, as well as age at listing, and living-related donor status. To account for population structure, we also included non-White race and Latino ethnicity as fixed terms that would not be removed from the model. The initial model was moderately predictive of recurrence (c-statistic 0.68, 95% CI 0.64-0.72). However, our final model was as predictive (c-statistics 0.68, 95% CI 0.64-0.71) but had an improved Akaike information coefficient (1098 versus 1102) and Bayesian information coefficient (1142 versus 1166). The final model included the recipient risk haplotype, B58, C3, DQ6, concordance at DQ7, and age at listing as explanatory variables.

**TABLE 3.** Multivariable models for the prediction of FSGS recurrence

	Initial model	Final model
	OR (95% CI)	OR (95% CI)
Race (Black = 1)	0.933	0.873
	0.661, 1.318	0.623, 1.222
Ethnicity (Latino = 1)	**0.648** *	**0.654** *
	**0.444,0.944**	**0.451,0.948**
Recipient risk haplotype	**1.569** *	**1.718** **
	**1.108, 2.224**	**1.231, 2.397**
Recipient DR52	0.802	
	0.585, 1.100	
Recipient B13	1.472	
	0.713, 3.040	
Recipient C3	**0.270** **	**0.270** **
	**0.112, 0.652**	**0.112, 0.650**
Recipient B58	0.551	**0.514** *
	0.283, 1.072	**0.265, 0.996**
Recipient DQ7	0.93	
	0.636, 1.359	
DQ7 match	**0.361** **	**0.334** ***
	**0.195, 0.670**	**0.193, 0.577**
Age at listing	**0.932** ***	**0.931** ***
	**0.901, 0.965**	**0.900, 0.964**
Related donor	1.258	
	0.856, 1.828	
Constant	1.482	1.371
	0.840, 2.615	0.819, 2.294

Bolded *P* are significant.

*P < 0.05

**P < 0.01

***P < 0.001.

CI, confidence interval; FSGS, focal segmental glomerulosclerosis; OR, odds ratio.

## DISCUSSION

This study represents the first analysis of HLA and FSGS disease recurrence following transplantation in pediatric patients. We demonstrate that certain HLA types are associated with either an increased or decreased risk of recurrence and identify an HLA risk haplotype that encompasses previously defined HLA antigens from NS genome-wide association study analyses. Finally, we show that concordance between donor and recipient HLA may additionally modify recurrence risk.

Multiple teams have sought to better understand the underlying pathophysiology of idiopathic NS and specifically FSGS. FSGS is defined on biopsy as segmental destruction of the glomerular capillaries and foot process effacement, providing insight into the structural changes that lead to loss of nephron function. However, full understanding of the environmental, immunologic, and/or genetic triggers that contribute to these destructive changes remain elusive. Familial clustering of FSGS spurred gene discovery studies that revealed a large number of pathogenic gene variants that cause monogenic FSGS; however, these only account for approximately 30% of steroid resistant nephrotic syndrome/FSGS cases.^27,28^ The majority of genes identified encode for proteins essential to the integrity^29^ and function of the glomerular podocyte.^30^ In contrast, the use of immunosuppressive agents has been shown to slow or mitigate disease progression in numerous cases of FSGS, suggesting an underlying immunological process for many patients.^31-37^ Experimental and clinical data show improvement of proteinuria after treatment with plasmapheresis, which supports the hypothesis that a circulating factor contributes to podocyte injury.^38^ Multiple candidate factors have been identified in blood including urokinase-type plasminogen activator receptor, cardiotrophin-like cytokine factor-1, anti-CD40 antibodies, and apolipoprotein A-Ib.^39-43^

HLA disease associations have been documented across numerous primary kidney disorders suggesting an early immunologic etiology for many patients evaluated for transplantation. Indeed, the 3 HLAs identified in our risk haplotype—HLA DR7, DR53, and DQ2—have been previously identified in multiple studies of primary NS.^15-18,20-24^ Additionally, all 3 of these HLAs and the risk haplotypes were associated with early recurrence, which is likely of more clinical concern than later recurrence. This may not be surprising given the central role of HLA molecules in initiating adaptive immune responses and their implication in many destructive autoimmune processes. There is evidence that class II HLA polymorphisms have evolved to offer maximal protection from pathogens at the expense of potential self-reactivity.^44^ In the present study, 4 HLAs were associated with increased risk of recurrent FSGS, with 3 being class II HLA, consistent with this theory. Our data, which utilize direct HLA typing (either serological typing or serological equivalents of molecular typing) and not single nucleotide polymorphism imputation extend the results of previous GWAS^20,21,24^ in a diverse cohort. This is important, as previous examination of single nucleotide polymorphism imputation of HLA has found errors, especially in non-White populations.^45^ Though there have been some small historical studies examining the relationship of serologic HLA type with primary NS and FSGS,^46,47^ we are the first to examine the association of HLA antigens in posttransplant FSGS recurrence.

One unique finding of this study is that HLA concordance between the recipient and kidney donor appeared to influence the FSGS recurrence risk. Whereas the conferment of risk was mediated only by the recipient HLA, the protective effect of HLA DQ7—associated with a decreased risk of recurrence—was determined by concordance with the donor. Though the mechanism by which concordance causes protection is unclear, it is possible that HLA DQ7 molecules are less likely to present peptides, which license immunologically mediated destruction leading to changes consistent with FSGS. Additionally, it is unclear if our inability to find this concordance effect more broadly is due to a true biological difference or mediation at the allelic and not the antigen level. Given that there are conflicting studies on the effect of donor factors in the risk of recurrence,^10-13^ further studies that specifically define HLA alleles based on their genetic sequence would be helpful in answering the clinical question of whether or not HLA concordance—of an allele or haplotype—leads to modifications in the risk of recurrent NS and FSGS.

More mechanistically intuitive is the fact that multiple class II HLA are associated with an increased risk of recurrence. HLA class II expression in the kidney is constitutively low, but is upregulated in response to inflammation and injury. Therefore, interaction between HLA class II and adaptive immunity generally involves the presentation of peptides in a proinflammatory environment. Indeed, studies to determine the expression of class II HLA in the kidneys of patients with FSGS may yield insights into the pathogenesis of the disease and the potential for recurrence.

Although our findings are important, our study has limitations. First, the data are retrospective from a large national registry. Reporting of the clinical event of recurrent FSGS may be incomplete; however, the generally high percentage of patients experiencing recurrence (28%) is compatible with known rates in the literature and not compatible with monogenic FSGS, which has a much lower recurrence rate.^14,48^ Also, all included patients are pediatric and do not have diabetes, limiting the chance of patients with secondary FSGS being included in our cohort. Our dataset does not contain diagnosis codes for other forms of NS such as minimal change disease; therefore, we may be excluding some patients inappropriately. The dataset also does not contain information regarding patient histology, known to be important in the clinical course of FSGS. Most importantly, the HLA data are reported from a wide variety of centers and are at the *antigen* level, whereas most genetic studies have found associations of HLA and FSGS at the *allele* level.^23^ This limitation may be a reason that we did not detect associations between all previously identified HLA and recurrent FSGS. However, our identification of a common risk haplotype DR7-DR53-DQ2 adds credibility to the associations we did find. Finally, it is possible that the associations we see here implicate not HLA in the pathogenesis of FSGS but rather other variants in linkage disequilibrium with those HLA.

In conclusion, we find that certain recipient HLAs are associated with risk of recurrent FSGS in pediatric kidney transplant recipients. Further prospective studies should be undertaken to validate these HLA as risk factors to further our understanding of the pathogenesis of FSGS and disease recurrence as well as to improve donor selection, and perioperative management for pediatric patients with FSGS.

## ACKNOWLEDGMENTS

The data reported here have been supplied by the Hennepin Healthcare Research Institute as the contractor for the SRTR. The interpretation and reporting of these data are the responsibility of the author(s) and in no way should be seen as an official policy of or interpretation by the SRTR or the US Government. We would like to acknowledge our funding from the National Institutes of Allergy and Infectious Disease (U01 AI152585-01) and the National Heart Blood and Lung Institute (R38HL143612-03).
